# Intensification in Genetic Information and Acquisition of Resistant Genes in Genome of *Acinetobacter baumannii*: A Pan-Genomic Analysis

**DOI:** 10.1155/2022/3186343

**Published:** 2022-12-27

**Authors:** Akhtar Ali, Ambrina Khatoon, Talat Mirza, Farah Ahmad

**Affiliations:** ^1^Department of Pharmacology, Ziauddin Medical College, Ziauddin University Karachi, Pakistan; ^2^Department of Molecular Medicine, Ziauddin Medical College, Ziauddin University, Karachi, Pakistan; ^3^Department of Research, Ziauddin Medical College, Ziauddin University, Karachi, Pakistan; ^4^Department of Community Health Sciences, Ziauddin University, Karachi, Pakistan

## Abstract

*Acinetobacter baumannii* (*A. baumannii*) attributes 26% of the mortality rate in hospitalized patients, and the percentage can rise to 46 in patients admitted to ICU as it is a major cause of ventilator-associated pneumonia. It has been nominated as the critical priority organism by WHO for which new therapeutic drugs are urgently required. To understand the genomic identification of different strains, antimicrobial resistance patterns, and epidemiological typing of organisms, whole-genome sequencing (WGS) analysis provides insight to explore new epitopes to develop new drugs against the organism. Therefore, the study is aimed at investigating the whole genome sequence of *A. baumannii* strains to report the new intensifications in its genomic profile. The genome sequences were retrieved from the NCBI database system. Pan-genome BPGA (Bacterial Pan-genome Analysis Tool) was used to analyze the core, pan, and species-specific genome analysis. The pan and core genome curves were extrapolated using the empirical power law equation *f*(*x*) = *a*.*xb* and the exponential equation *f*1(*x*) = *c*.*e* (*d*.*x*). To identify the resistant genes with resistant mutations against antibiotics, ResFinder and Galaxy Community hub bioinformatics tools were used. According to pan-genome analysis, there were 2227 core genes present in each species of the *A. baumannii* genome. Furthermore, the number of accessory genes ranged from 1182 to 1460, and the unique genes in the genome were 931. There were 325 exclusively absent genes in the genome of *Acinetobacter baumannii.* The pan-genome analysis showed that there is a 5-fold increase in the genome of *A. baumannii* in 5 years, and the genome is still open. There is the addition of multiple unique genes; among them, genes participating in the function of information and processing are increased.

## 1. Introduction

A nonfermenter Gram-negative coccobacillus known as *A. baumannii* was thought to be a low category pathogen in past decades but now has arisen as a foremost cause of community and hospital-acquired infections [1]. It can survive in any environment for longer durations, and it is found to be resistant to many classes of antibiotics and disinfectants [2]. *A. baumannii* has been identified as a responsible factor that causes pneumonia and septicemia in immunocompromised individuals. It confers 26% of mortality rate in hospitalized patients, and the percentage can rise to 46 in patients admitted in ICU as it is a major cause of ventilator-associated pneumonia [3, 4]. It has been categorized among ESKAPE (*Enterococcus faecium, Staphylococcus aureus, Klebsiella pneumoniae, Acinetobacter baumannii, Pseudomonas aeruginosa*, and *Enterobacter* species) pathogens. WHO has kept carbapenem-resistant *A. baumannii* (CRAB) as the topmost priority organism for which new therapeutic drugs are urgently required; therefore, rigorous antibiotic stewardship and infection control measures should be taken to control the MDR infections and make it a presenting issue for new therapeutic options [5].

**Figure 1:**
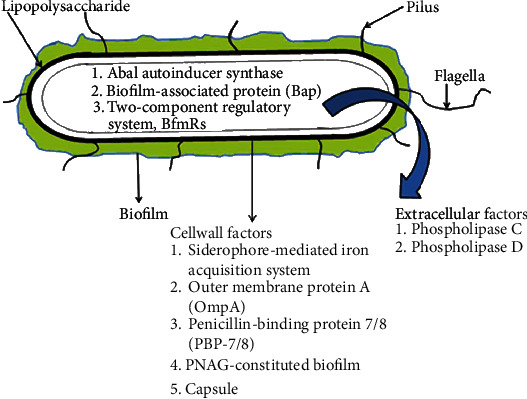
Virulence factors of *A. baumannii* [6].

It has been suggested that quorum sensing (QS) is involved in the synthesis of multidrug-resistant biofilm, one of the major virulent factors of *A. baumannii*. This multidrug-resistant biofilm provides insulation to organisms that help them in surviving the harsh environment.  Figure 1 shows the virulent factors of *A. baumannii* [6, 7]. Furthermore, genomic plasticity provides the ability for *A. baumannii* to survive on abiotic surfaces. Acquiring genes by horizontal gene transfer and through membrane vesicles has contributed to the development of MDR and XDR strains of these virulent bacteria [8–10].

Figure 1 demonstrates the variety of virulence characteristics that enable *A. baumannii* to tenaciously resist environment, form biofilms, migrate, interact with host cells, and secrete proteins.

Whole-genome sequencing (WGS) provides deep knowledge regarding species identification, antimicrobial resistance, and epidemiological typing of organisms to researchers. It helps in the identification of new drug targets and the development of new drugs [11]. The ability of WGS to distinguish the strains that differ by even a single nucleotide provides high-resolution information [12]. It has been documented that the *A. baumannii* possesses a dynamic genome, and it has been postulated that the genomic sequence of this virulent organism may vary in different regions of the globe [13, 14]. The aim of the study was to analyze the genomes of *A. baumannii* using pan-genomic techniques and to generate significant data on its genomic characteristics on different variants.

## 2. Methodology

Pan-genome BPGA (Bacterial Pan-genome Analysis Tool) was used to analyze the core, pan, and species-specific genome analysis. A total of 50 complete genomes were retrieved from the NCBI database, and 2 gbk files were used as input files in the BPGA pipeline. Furthermore, genome assembly and annotation reports were opted to reach the genomic sequences. Then, sequence files were downloaded from the database (GenBank). Usearch tool was applied to develop clusters. The pipeline was used for 99% sequence identity as the cut off value for further analysis. Additionally, cluster of orthologs (COGs) and KEGG pathway detection was carried out by using the BPGA tool. Then, phylogeny based on core and pan proteome was explored using COG distribution, KEGG pathway analyses, and COG distribution. Furthermore, the retrieved data of genomes of *A. baumannii* were analyzed with and in addition mass screening of antimicrobial resistance, and virulence gene was conducted by ABRicate software [15, 16] to get the information regarding acquired resistant genes and their resistance against specific antibiotic drugs.

Central genome is crucial in each species, and it is responsible for housekeeping of cell, but the axillary genome carries the genes of resistance, stress, and pathogenesis.

## 3. Results and Discussion

The functional adaptations of *Acinetobacter baumannii* showed *B*_pan_ values of 0.169136 (i.e., <1), suggesting that the pan-genome is still open but may be closed soon. It highlights that to combat the environmental stress, *A. baumannii* has gone through various evolutionary changes (Table 1). Furthermore, the pan-genome analysis also accentuated that the number of accessory gene families increases as the genome size increases. A total of 2227 core genes were present in all strains; whereas there were 1301 ± 61 number of accessory genes in the genome. There were 325 exclusively absent genes in the genome of *A. baumannii*; moreover, there were 931 unique genes present in the genome, and the genome no. 1 has acquired the highest number of unique genes, that is, 153 as shown in Table 2.

**Figure 2:**
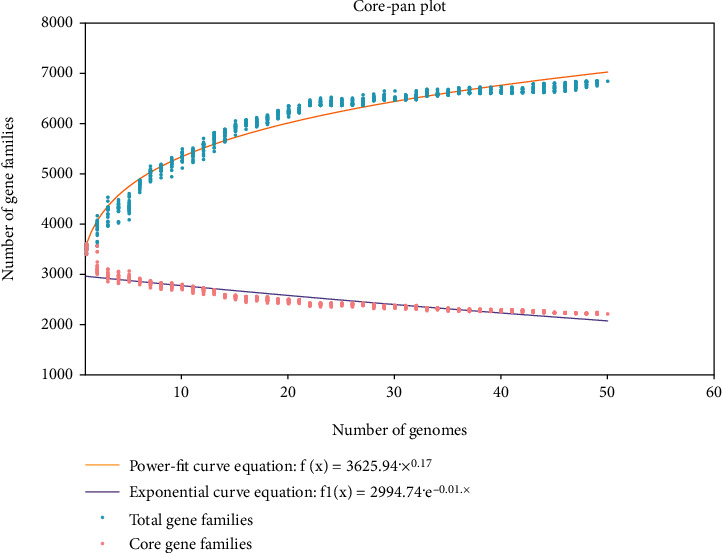
The pan and core genome plot of *A. baumannii* genomes.

**Figure 3:**
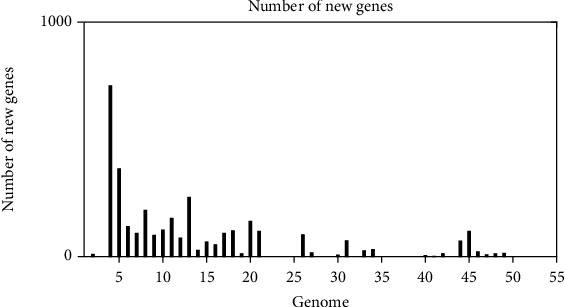
Number of new genes contributing to pan-genome of *Acinetobacter baumannii.*

**Figure 4:**
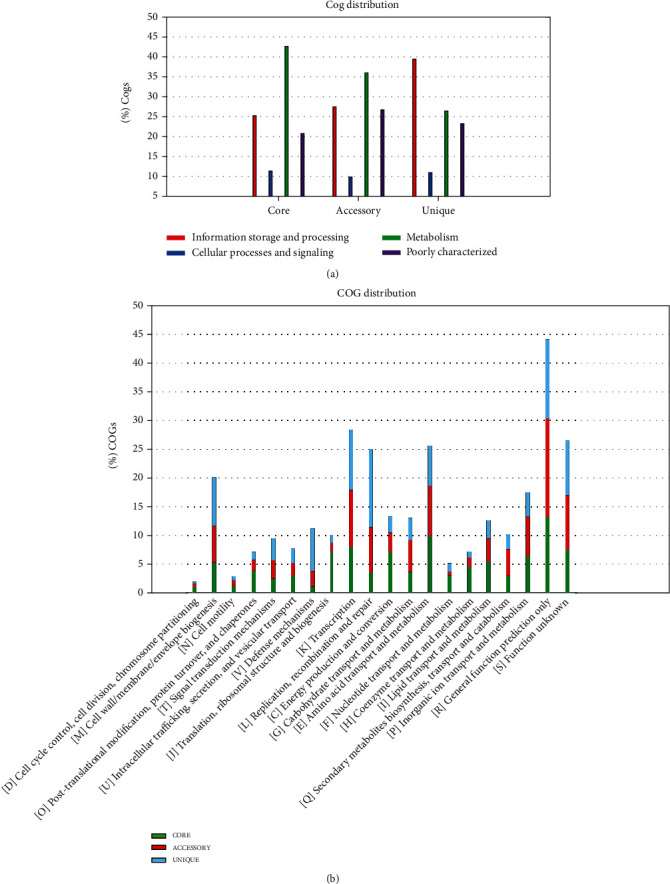
(a) COG distribution of the genes making up the core, accessory, and unique portion of 164 the *A. baumannii* genome. (b) Functional distribution of core, accessory, and unique genes.

The plateau formation on the pan-genome indicated that the genome is still open and will be closed soon. The pan-genome of bacteria including *A. baumannii* should be kept open permanently due to its evolution and horizontal gene transfer as highlighted in core pan plot (Figure 2). The new genes that contribute to the highest number in pan-genome of *A. baumannii* were 931 (Figure 3). COG analysis showed that the core and accessory genes were involved in the function of metabolism prominently; however, unique genes were found to be involved in the function of information storage and processing (Figures 4(a) and 4(b)).

**Figure 5:**
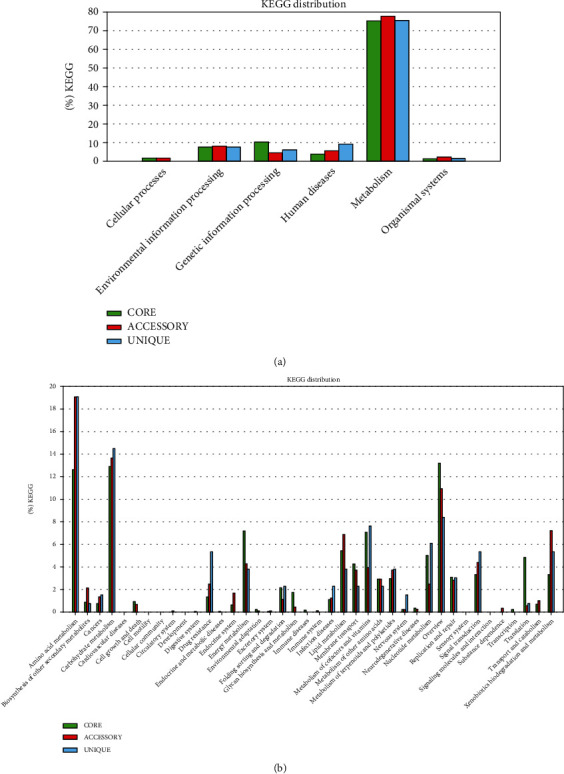
(a) KEGG distribution of core, accessory, and unique genes. (b) KEGG analysis, functional distribution of core, accessory, and unique genes in *A. baumannii* genome.

Moreover, comparative KEGG analysis functionally distributed the core, accessory, and unique genes and highlighted that these genes were mostly responsible for the human diseases, genetic information, and environmental adaptations as shown in Figures 5(a) and 5(b).

The *A. baumannii* definitely be categorized as an emerging pathogen of clinical significance. It has gained the attention of scientists in recent years; increase in the size of its genome and resistance to multiple drugs are making this organism more virulent, particularly in hospital settings [17]. The increase in the occurrence of infections due to the vulnerability of this organism is disturbing healthcare settings as well as stroking a high economic burden [18]. In order to understand its metabolic processes and resistance profile, study of its genome and study of variations in reported genetic patterns from different countries is required. The data from these studies will facilitate the researchers to target the *A*. *baumannii* according to its genetic identification and will be beneficial to overcome the resistance [19, 20]. In this manuscript, we have highlighted the current pan-genomics of *Acinetobacter baumannii.* Furthermore, we also accentuate the distribution of its genes according to the metabolic processes (i.e., COG and KEGG analyses) involved in its genome. Since 2011 studies have been done on the pan-genome analysis of *A. baumannii* initially six and seven, and in 2016, thirty genomes were studied; however, now in 2022, the number of genomes has been increased at the NCBI website that we have analyzed in the current study [21, 22]. The information of the genome of an organism provides the details of its genetic evolution; it highlights the increase in pan-genome size which eventually indicates that the genome is still open and increasing. According to pan-genome analysis, its genome has been found open which is indicating that the genome can still acquire more genes and environmental adaptations and can lose certain genes, that is, further strengthening its genomic evaluation and vulnerability [23, 24].

The pan-genome of *A. baumannii* seemed to be composed of 37254 genes; out of which, 36323 genes were formulating the core genome, 931 were unique genes, and 301 genes were found to be absent in the current analysis. Compared to the analysis of Hassan et al., the current genome has increased 5-folds in five years [21]. The increase in the size of genome among prokaryotes including *A. baumannii* is attributed to horizontal gene transfer; it has been identified that due to the absence of sexual reproduction, bacteria evolve through conjugation that facilitates gene transfer among different strains of organisms [25]. This gene transfer increases the size of the genome and resistance profile in the organisms that have not encountered the particular antibiotic before, but the gene of resistance was transferred to it from the bacterial strain which was exposed to that antibiotic earlier [26]. The COG distribution analysis (Figure 4(a)) revealed that among core genes in the new genome of fifty plasmids, the higher percentage observed was of genes involved in performing the function of metabolism; however, in unique genes, the major proportion was obtained by genes which attribute function of information and processing in the genome. This is highlighting that the evolution in the genome particularly the addition of unique genes is increasing the information that is thought to be a major factor contributing an increase in resistance against multiple antimicrobial drugs [27].

The KEGG analysis of the *A*. *baumannii* highlighted the functional distribution of core, assessor, and unique genes of its genome. As depicted in Figure 5(b), it was identified that there was more adaptation of accessory and unique genes that were used for the metabolic processes including metabolism of amino acids, carbohydrates, vitamins, and nucleotides. Additionally, this analysis also revealed that among the drug resistance, there was highest acquisition of unique genes highlighting the potential of this organism to become one of the most resistant bacteria. Similar to the data reported by Xiong et. al., the KEGG analysis of the current study also emphasized that there was more functional adaptation in the genes of amino acid metabolism; however, in their study, they knocked out the *abal* to evaluate the resistant profile considering it as the most important virulence factor; however, they could not found any change as there was no difference in virulence as well as resistance profile of the organism that in perspective of the current study may be due to acquisition of unique genes which are responsible for drug resistance [28].

**Figure 6:**
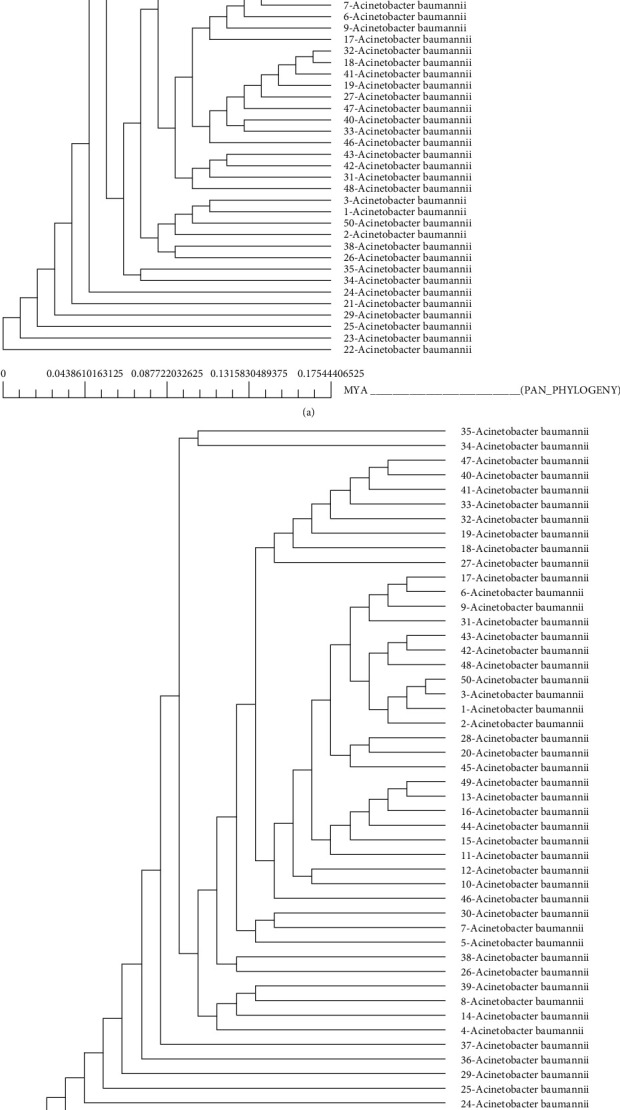
(a) Pan-genome phylogenic tree. (b) Core genome phylogenic tree.

The phylogenic trees were formulated to compare the evolution of the pan and core genomes of *A. baumannii*. Figures 6(a) and 6(b) depict the phylogenic trees of the pan and core genomes.

The resistance acquisition was analyzed by ResFinder software, and mass screening of resistant genes was evaluated by ABRicat tool. According to the findings of ResFinder, the genome of *A. baumannii* seemed to be resistant against all antibiotic drug classes including beta lactams, aminoglycosides, fluoroquinolones, and sulfonamides. However, the ABRicat analysis revealed that all the antibiotic drugs belonging to different classes can come out to be ineffective, as the genome of *A. baumannii* has acquired mutant genes possessing resistance against conventionally used antibiotic drugs.  Table 3 shows resistant genes against available therapeutic options.

The phylogenic analysis of this bacteria showed that the strains belonging to the same clonal lineages have strong similarities in their proteome [29]. The phylogenic analysis has also shown that numerous similar proteins are expressed by various strains of *Acinetobacter baumannii*. Therefore, in addition to studying the genome of this pathogen, the paradigm has shifted to examining the structure of similar proteins expressed by the genes of various strains of *A. baumannii* to discover new epitopes that could aid in the creation of vaccines against this vulnerable organism [30, 31]. Growth in the new genome of different organisms is increasing the lethality of bacterial diseases; the lethality is attributed to resistance against therapeutic drugs. Resistance against multiple antibacterial drugs is increasing day by day; previously, it was thought that irrational prescription, suboptimal doses, and patient incompliance are the factors involved in the development of resistance among microorganisms, but in the current era, genomic evolution and characterization are adding more information in it which is highlighting that the increase in resistance is not only accredited with prescription of drugs, but the genetic growth, acquisition of different genes from other organisms, and strains via horizontal gene transfer are some of the major contributing factors in the development of the multidrug-resistant organisms [32–34]. The ResFinder and Galaxy Community hub bioinformatics tools highlighted that the genome of *A. baumannii* has developed resistant to all available antibiotics, and the gene acquisition is in process as the genome is still open. That is an alarming signal which is suggesting the creation of superbug that cannot be killed by any available antibiotic [35]. The current need to deal with the persistent scenario of increase in the antibacterial resistance is identification of mechanisms by which the bacteria are protecting themselves; along with this, identification of mode of acquisition of genes is also required so that further expansion of genome in terms of acquiring resistant genes from other bacteria can be inhibited.

## 4. Conclusion

The pan-genome analysis showed that there is a 5-fold increase in the genome of *A. baumannii* in 5 years, and the genome is still open. There is the addition of multiple unique genes; among them, genes participating in the function of information and processing are more increased. The phylogenic analysis is revealing homology among different strains of this organism. Furthermore, the ResFinder and Galaxy Community hub bioinformatics tools revealed that the genome of *A. baumannii* has been resistant to almost all available antibiotics, so there is a critical requirement for the development of novel therapeutic agents against this organism.

## Figures and Tables

**Table 1:** Pan-genome statistics.

	Pan-genome	Core genome
Fit law	Power	Exponential
Equation	*f*(*x*) = *a*.*x*∧*b*	*f*1(*x*) = *c*.*e*∧(*d*.*x*)
Parameters	*a* = 3625.94	*c* = 2994.74
__________	*b* = 0.169136	*d* = −0.00720414
Expected size	6846	0
Estimated size	7027.12	2088.93

**Table 2:** Number of unique genes present in different species of *A. baumannii.*

Genome number	Gene ID	No. of accessory genes	No. of unique genes	No. of exclusively absent genes
1	NZ_CP015121	1289	0	2
2	NZ_CP045110	1289	1	1
3	NZ_CP059040	1282	0	7
4	NZ_CP046654	1450	153	8
5	NZ_CP059300	1372	21	6
6	NZ_CP009257	1337	28	11
7	NZ_CP018254	1342	55	5
8	NZ_CP027611	1460	6	0
9	NZ_CP050385	1314	22	5
10	NZ_CP033869	1279	63	9
11	NZ_CP048849	1281	94	19
12	NZ_CP014528	1211	56	78
13	NZ_CP022283	1360	36	4
14	CP027528	1367	13	18
15	CP040259	1223	21	3
16	NZ_CP050403	1327	24	7
17	NZ_CP041035	1304	63	30
18	NZ_CP023020	1344	0	1
19	NZ_CP021326	1329	0	5
20	NZ_CP060732	1305	1	0
21	NZ_CP051869	1350	0	0
22	NZ_CP072280	1350	0	0
23	NZ_CP072295	1350	0	0
24	CP072275	1351	0	0
25	NZ_CP072290	1349	0	0
26	NZ_CP072300	1260	0	0
27	NZ_CP027178	1284	14	19
28	NZ_CP045528	1306	0	0
29	NZ_CP051875	1349	0	0
30	NZ_CP072270	1329	5	1
31	NZ_CP026338	1192	27	28
32	NZ_CP060029	1345	0	0
33	NZ_CP010779	1239	1	3
34	NZ_CP013924	1301	0	0
35	NZ_CP040050	1302	0	0
36	NZ_CP040050	1338	0	0
37	NZ_CP072398	1339	0	0
38	NZ_CP072398	1246	0	3
39	NZ_CP027183	1334	0	6
40	NZ_CP020597	1279	4	2
41	NZ_CP014215	1332	1	5
42	NZ_CP059354	1182	0	0
43	NZ_CP059729	1182	0	0
44	NZ_CP059729	1273	64	7
45	NZ_CP012952	1199	108	4
46	CP044519	1237	18	11
47	NZ_CP024576	1259	7	5
48	NZ_CP050523	1194	11	5
49	NZ_CP039341	1285	14	7
50	NZ_LS483472	1288	0	0

**Table 3:** Resistant genes against available therapeutic options for *A. baumannii* (ResFinder and ABRicat analyses).

Resistant gene	Accession no.	ResFinder	ABRicat	Coverage
aac(3)-Ia	X15852	Aminoglycoside resistance	Gentamicin	87.07865169
aac(3)-IIa	CP023555	Aminoglycoside resistance	Gentamicin; tobramycin	100
aac(6′)-Ian	AP014611	Warning: gene is missing from note files. Please inform curator.	Amikacin; tobramycin	100
aac(6′)-Ibcr	EF636461	Fluoroquinolone and aminoglycoside resistance	Streptomycin	100
aac(6′)-Ib-Hangzhou	FJ503047	Warning: gene is missing from note files. Please inform curator.	Streptomycin	100
aadA1	JQ480156	Aminoglycoside resistance	Streptomycin	100
aadA24	DQ677333	Aminoglycoside resistance	Gentamicin; tobramycin	98.97435897
aadA2b	D43625	Aminoglycoside resistance	Streptomycin	100
ant(3^″^)-Ia	X02340	Warning: gene is missing from note files. Please inform curator.		81.48148148
aph(3′)-Ia	X62115	Aminoglycoside resistance	Streptomycin	100
aph(3^″^)-Ib	AF321550	Aminoglycoside resistance alternate name; aph(3^″^)-Ib	Streptomycin	100
aph(3′)VIa	X07753	Aminoglycoside resistance	Amikacin	100
aph(6)-Id	M28829	Aminoglycoside resistance alternate name; aph(6)-Id	Streptomycin	100
armA	AY220558	Aminoglycoside resistance	Streptomycin	100
ARR-2	HQ141279	Rifampicin resistance	Streptomycin	100
blaADC-25	EF016355	Beta-lactam resistance	Amikacin; gentamicin; tobramycin	100
blaCARB-14	JQ364968	Warning: gene is missing from note files. Please inform curator.		100
blaCTXM-124	JQ429324	Beta-lactam resistance		100
blaCTXM-15	AY044436	Beta-lactam resistance alternate name; UOE-1	Ampicillin; amoxicillin; piperacillin	100
blaNDM-1	FN396876	Beta-lactam resistance	Amoxicillin; ampicillin; aztreonam; cefepime; cefotaxime; ceftazidime; ceftriaxone; piperacillin; ticarcillin	100
blaOXA-235	JQ820240	Beta-lactam resistance	Amoxicillin; amoxicillin+clavulanic acid; ampicillin; ampicillin+clavulanic acid; cefepime; cefixime; cefotaxime; cefoxitin; ceftazidime; ertapenem; imipenem; Meropenem; piperacillin; piperacillin+tazobactam	100
blaOXA-239	JQ837239	Beta-lactam resistance		100
blaOXA-371	AB871653	Beta-lactam resistance	Amoxicillin; +clavulanic acid; ampicillin; +clavulanic_; ceftazidime; piperacillin	100
blaOXA-402	JX865391	Warning: gene is missing from note files. Please inform curator.		100
blaOXA-64	AY750907	Beta-lactam resistance	Imipenem; meropenem	100
blaOXA-65	AY750908	Beta-lactam resistance	Amoxicillin; ampicillin; cefoxitin; imipenem; meropenem; piperacillin; piperacillin+tazobactam	100
blaPER-1	GU944725	Beta-lactam resistance		100
blaPER-7	HQ713678	Beta-lactam resistance	Amoxicillin; ampicillin	100
blaTEM1A	HM749966	Beta-lactam resistance alternate name; RblaTEM-1		100
catA1	V00622	Phenicol resistance	Amoxicillin; ampicillin; cephalothin; piperacillin; ticarcillin	100
catB8	AF227506	Phenicol resistance	Chloramphenicol	100
cmlA1	M64556	Phenicol resistance	Trimethoprim	99.91769547
dfrA1	X00926	Trimethoprim resistance	Chloramphenicol; florfenicol	100
floR	AF118107	Phenicol resistance	Erythromycin	100
Mph(E)	DQ839391	Macrolide resistance		100
Msr(E)	FR751518	Macrolide, lincosamide, and streptogramin B resistance	Sulfamethoxazole	100
qacE	X68232	Disinfectant resistance	Doxycycline; tetracycline	100
sul1	U12338	Sulphonamide resistance	Doxycycline; tetracycline; minocycline	100
Tet(A)	AJ517790	Tetracycline resistance	Doxycycline; tetracycline	100

## Data Availability

The data were collected from the NCBI GenBank, as it was a study based on the use of bioinformatics tools.
